# Identifying and Reengaging Patients Lost to Follow-Up in Rural Africa: The “Horizontal” Hospital-Based Approach in Uganda

**DOI:** 10.9745/GHSP-D-18-00394

**Published:** 2019-03-22

**Authors:** Faraz Alizadeh, Gideon Mfitumuhoza, Joseph Stephens, Christopher Habimaana, Kwiringira Myles, Michael Baganizi, Gerald Paccione

**Affiliations:** a Boston Children's Hospital, Boston Medical Center, and Doctors for Global Health, Boston, MA, USA.; b Kisoro District Hospital and Doctors for Global Health, Kisoro, Uganda.; c Montefiore Medical Center, Albert Einstein College of Medicine, and Doctors for Global Health, Boston, MA, USA.

## Abstract

Among the many challenges facing health systems grappling with the explosive growth of chronic disease in Africa are continuity of care, particularly in poor, rural areas. We report the strategy, field experience, and results of an ongoing 6-year follow-up program operating in a rural district hospital in Kisoro, Uganda, that attempts to locate and reengage patients lost to follow-up (LTFU) from communities that are largely without phones, addresses, or paved roads. The program works with diverse hospital clinics, including chronic diseases, HIV, tuberculosis (TB), nutrition, and women's health, to identify patients who have not returned to care, employing a modest staff who spend about 20 days monthly making outreach visits by motorcycle in search of approximately 130 patients. We describe the organization of this unique “horizontal” program and report on follow-up outcomes between November 2015 to October 2016. Between 30% and 60% of patients were found to have lapses in care. The follow-up program was able to locate 64% of patients, with a reengagement rate of 54% to 92% (average, 69%) depending on the clinic. The program costs approximately US$5 per patient LTFU but about US$40 per patient maintained in care. The hospital-based follow-up program that cuts across diverse clinics and wards was novel and feasible in this rural sub-Saharan African setting.

## Abstract (precis)

Between 30% and 60% of hospital outpatient clinic patients were lost to follow-up. A defaulter-tracking service using performance-based remuneration for outreach workers, cutting across different clinical services, improved patient retention overall but varied by disease, with the poorest outcomes among patients with HIV.

## INTRODUCTION

While adherence to medication is a challenge for patients with chronic disease everywhere, it's particularly problematic in low- and middle-income countries (LMICs) where the disease burden is great and growing and access to care is most limited. Treatmentof tuberculosis (TB), HIV, noncommunicable diseases (NCDs), and malnutrition are fraught with attrition, undermining disease control.

In 2015, there were 10.4 million new cases of TB worldwide. Although the World Health Organization (WHO) reported a 4% lost to follow-up (LTFU) rate globally (with only 9% of countries reporting >15% LTFU), these data are at odds with experience in the field and there is growing concern over the accuracy of population-level estimates.^1^ A recent report from Uganda provides a more sober picture: only 66% of patients with TB and HIV coinfection living in rural areas completed TB therapy compared with 81% of urban dwellers.^2^^,^^3^ HIV affects an estimated 37 million people globally, of whom 70% live in sub-Saharan Africa. Two meta-analyses of tracing programs for patients with HIV in LMICs revealed LTFU rates of 17% to 29% at 24 months.^4^^–^^6^ In terms of NCDs, they are already a leading cause of morbidity and mortality in LMICs, with 74% of the 38 million annual NCD deaths occurring in LMICs and over 80% of deaths considered “premature.”^7^ Reports from various LMICs reveal a 22% to 42% LTFU rate at 6 years for patients with hypertension, a 35% LTFU rate for patients with diabetes, and 27% to 34% LTFU rate at 1 year for patients with epilepsy.^8^^–^^12^ The literature on malnourished children LTFU from nutrition programs^3^^,^^13^ and women LTFU after screening positive for cervical cancer paint a similar picture.^14^^–^^17^

Although there are some reports of disease-specific programs for HIV or TB that address the LTFU issue,^3^^,^^13^^,^^18^^–^^22^ there have been no descriptions of hospital-wide initiatives that routinely follow patients in rural communities in low-income countries. In this article, we describe a strategy to maintain in care patients from various outpatient clinics of a remote, rural district hospital in Kisoro, Uganda. The experience of this follow-up program is germane to both clinicians and researchers trying to improve outcomes for long-term care in rural Africa.

The southwest Ugandan district of Kisoro is poor and rural with 86% of the population earning US$1–2 each day as subsistence farmers.^23^ There are only two hospitals in the district of nearly 300,000 population, one public—Kisoro District Hospital (KDH)—and the other private, and there was 1 doctor per 40,000 people in the Kisoro district at the time of this study. Due to a paucity of trained medical personnel, in 2005 KDH partnered with a U.S.-based NGO (Doctors for Global Health) and a U.S. academic medical center (Montefiore Hospital/Albert Einstein College of Medicine) to help staff the inpatient adult medicine wards. Through this collaboration, the Chronic Care Clinic (CCC) was started in 2006. The mission of the CCC is to provide continuity of care to patients with chronic disease. The CCC was the first chronic disease clinic in Southwestern Uganda and the only institutional source of free continuous care in the district. The need for ongoing monitoring and daily medications for chronic disease was largely unrecognized among the rural population at the time the clinic was founded, a situation compounded by lack of experience in chronic disease management among KDH's novice and ever-changing providers. Local surveys indicated that less than 10% of the clinic population could afford or would be willing to buy medications for chronic disease management in local pharmacies or the private hospital, with almost all such “affluent” clients living within Kisoro town proper. Patients with NCDs were identified on the inpatient wards and given CCC appointments on discharge. However, many of these “ward discharges” failed to return.

Discontinuing therapy was also a majorproblem among patients newly diagnosed with TB, and the local TB program did not have the funding or personnel to contact patients at home. Adherence to TB medications is not supported by directly observed therapy (DOT) in Kisoro, but rather by patient self-recording of drug ingestion, possibly with family assistance. DOT had never been established due to funding shortages, but for some years prior to 2010 the Global Fund to Fight AIDS, Tuberculosis and Malaria provided money for “family treatment supporters” who would identify and coach a family member to deliver and document treatment. When the funding ended in 2010, so did the program, replaced only by a monitored schedule of drug pickup at KDH or a local health center at specified intervals. If the patient did not pick up the medication from the hospital or health center, it was recorded—but tracing the patient in the community was not possible until KDH established the follow-up program.

Access to phones in the community was low (and still is low but improving), making it difficult to locate the patients. In 2018, for example, only about half of KDH patients had phone access, either personal or family, and most of the time the phones were off or not charged.

In 2012, the KDH follow-up program was initiated, run by 1 staff member on a motorcycle, to locate ward discharges and patients with TB who were lost to follow-up, attempt to reengage them in care, and document outcomes. As the program matured, it started to also follow patients with HIV and long-term CCC enrollees who were LTFU. In October 2015, a coordinator and additional field assistants were hired and patients who screened positive for cervical cancer in the women's clinic and malnourished children who were LTFU incorporated. Thus, the KDH follow-up program, which began as a side project, became embedded in the larger hospital system as an integral component of multiple clinical services.

The Kisoro District Hospital follow-up program was started in 2012 and is currently an integral component of multiple clinical services.

Since a major determinant of successful follow-up with health services is the cost borne by the patient, it should be emphasized that all health services and medication costs are free at KDH and in the public health sector of Uganda generally. However, drugs are often not in stock at these facilities. With the support of WHO, the U.S. President's Emergency Plan for AIDS Relief (PEPFAR), and other international funding initiatives, HIV and TB medications are usually in stock, but drugs for NCDs such as hypertension and diabetes are available only 50% to 80% of the time depending on drug and month. When the drugs are unavailable, KDH outpatients are asked to purchase the medications in local pharmacies with vouchers, supported by Doctors for Global Health, that cover 60% of the cost.

## PROGRAM DESCRIPTION AND METHODS

### Feeder Clinics and Geographical Sets

The KDH follow-up program traces patients LTFU from 6 hospital units or “feeders”: ward discharges, the inpatient TB registry, and 4 hospital clinics (comprising the HIV, CCC, nutrition, and women's clinics). Although follow-up results from the women's clinic have been within the range of the other feeder clinics, data were lost due to computer mishaps, and so its results are not included in this article.

Lists of patients LTFU from each feeder are submitted and patient locations entered into 1 of 9 geographically organized “sets” of 8–15 villages, each served by a common district road. Once a set has at least 8 patients LTFU, the follow-up coordinator first reconfirms with the feeders that the identified patients have not returned to the clinic recently, and a staffer sets off to locate and follow-up with the patients. The follow-up team meets with the various feeder clinics 1 to 2 times monthly to report on outcomes of the follow-up efforts and resolve any problems. Approximately 90% of patients seen at KDH live within Kisoro District and qualify to be enrolled in the follow-up outreach should they become LTFU.

### Follow-Up Procedures

The follow-up staff members consult the village chairman, the community health worker, or others in the community to help locate the patient. To maintain confidentiality, if asked (a rare occurrence), the staff members say that they are carrying a message from a hospitalized friend. If the patient is not home, a message to phone is left with the family. If no return call is or can be made, the follow-up staff member makes a second visit. If family members want details, none are provided, and the staff member explains that the message to contact was relayed by hospital personnel.

If the patient is located, the staff member first administers a brief disease-specific survey inquiring about reasons for not returning to the clinic and then discusses important aspects of the disease emphasizing the role of continuous care. Patients are encouraged to return and given an appointment and a note to provide to the clinic staff. For patients who do not return to KDH after being contacted and referred, a second outreach is made only for patients with TB due to the disease's public health consequences.

### Staff and Stipend System

The KDH follow-up program employs 1 full-time coordinator and 3 part-time assistants. All are university graduates though not in the health care field. All of the assistants have other roles in the hospital or district (as a CCC coordinator, environmental officer, and social worker). The program averages 20 follow-up days monthly, or 1–2 outreach days in the field weekly per staff member. At current capacity, the system has the potential to make approximately 150 patient visits per month and averages 130 follow-up visits.

**Figure:**
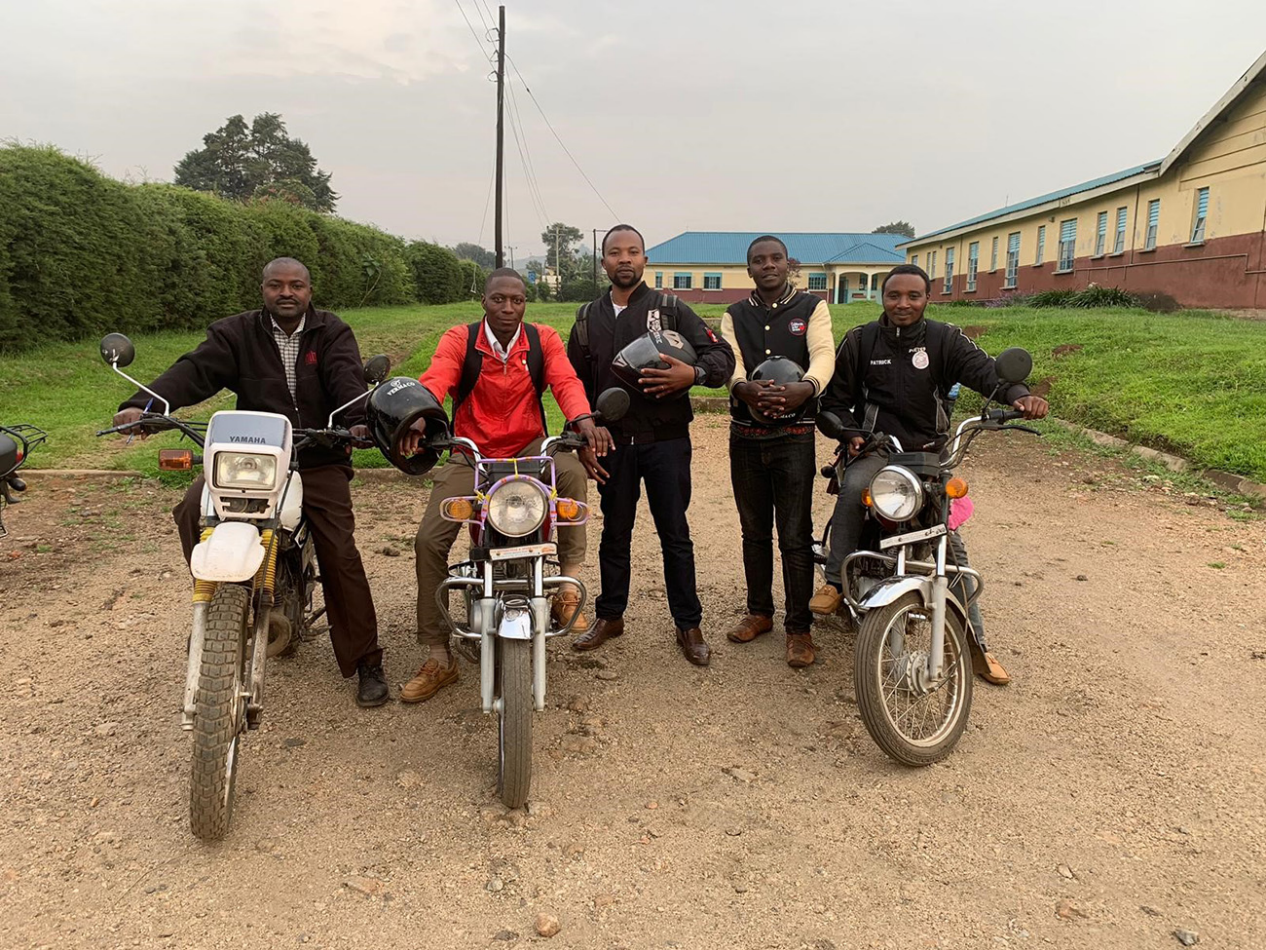
The Kisoro District Hospital follow-up team (including authors Gideon Muhoza and Christopher Habimana) use motorcycles to locate patients lost to follow-up in their communities. © 2019 Charles Moon/Doctors for Global Health

After a year of generally lackluster (but individually variable) success locating patents, a point-based stipend system was instituted to motivate the field workers to find and interview patients, or if that proved impossible, to determine what happened to them. The field workers earn 2 points for each patient they find and interview, 1 point for contacting the family, or 0.5 point if they cannot locate the patient. They earn 100% of their daily salary when they accumulate 10 points. Thus, with 8 patients in a set, each field worker can potentially make 160% of his usual salary if they find and interview each patient. If a field worker scores less than 10 points on a particular day (which is rare), he would get a lower salary for that day.

The hospital uses a performance-based system to motivate field workers to locate patients lost to follow-up.

### Definition of Terms

Loss to follow-up was defined differently by each of the program's 6 feeders, depending on treatment goal (curative or chronic-indefinite), the clinical or public health implications of foregoing treatment, and the feasibility of tracking patients for the clinic (Table 1). For example, ward discharge patients were considered LTFU if it had been 1 month or more since their last clinic appointment; 1 month was deemed the approximate time when patients' medications would be depleted or their clinical condition would start to deteriorate. In contrast, the LTFU time period for TB clinic patients was 2 or more weeks because patients are scheduled to pick up their medications every 2 weeks, with significant public health implications when there are breaks in treatment. Given the large numbers potentially requiring follow-up and the program's limited capacity, disease-specific severity criteria (not shown) were incorporated to define CCC patients LTFU. For example, for patients with “severe” NCDs, the LTFU period was 3 months, whereas for those with “moderate” severity, the LTFU period was 6 months. It should be noted that some patients identified as LTFU by the KDH follow-up program may actually have been receiving suitable follow-up from another service provider, but this was probably rare given the lack of providers and the poverty of the population.

**TABLE 1.** Lost to Follow-Up Defined at Kisoro District Hospital, Uganda, by Hospital Unit

Hospital Unit	LTFU Definition	Rationale	Frequency of Chart Review
Ward discharge	Missed first CCC appointment by 1 month	Approximate time before clinical deterioration and/or depletion of medications.	Weekly
Inpatient TB registry	Missed drug refill appointment by 2 or more weeks	Patients pick up medication every 2 weeks; public health implications for breaks in treatment are significant.	Monthly^a^
HIV clinic	Missed 2 monthly appointments (either pre- or post-ART initiation)	Although patients are scheduled to pick up medications monthly, many come 1 or 2 weeks post-appointment, so a 2-month interval captures the late-comers.	Every 2 weeks
Chronic Care Clinic	Patient with at least 2 prior visits (i.e., regular CCC patient) who has not returned for 3–6 months, depending on disease severity (3 months for most severe 25% of patients, 6 months for less severe)	Risk severity stratification applied due to large number of CCC patients and limited outreach capacity.	Every 2 months
Nutrition clinic	Missed 1 appointment	Low threshold applied due to population of vulnerable children.	Monthly

Abbreviations: ART, antiretroviral therapy; CCC, Chronic Care Clinic; LTFU, lost to follow-up; TB, tuberculosis.

a TB patients identified as LTFU could be off their medications for more than 1 month since staff identify TB patients LTFU once a month.

In addition to assessing LTFU rates, we also assessed lapses in care from the CCC and HIV clinics, using an interval of 3 months (without severity criteria) to define lapse from the CCC clinic and 2 months to define lapse from the HIV clinic. These proportions reflect the general level of appointment adherence we could expect in our rural district hospital among *all* patients. Thus, lapse proportions are based on an inception cohort of “all-comers” to the CCC and HIV clinics over a defined period of time, whereas LTFU rates are based on selected groups of patients meeting various clinically pragmatic inclusion criteria. We measured rates of 3-month lapse from CCC care or 2-month lapse from HIV care for both new enrollees (incidence cohort) and existing clinic patients (prevalence cohort) between May 2015 and April 2016. For the incidence group, a lapse counted as any such period in their first post-enrollment year, thus extending well into 2017 for those who enrolled in 2016. Inclusion criteria for *active* “prevalence” patients were at least 3 CCC or HIV clinic visits before May 2015 with at least 1 visit between January and April 2015, or, if they first enrolled in early 2015, returning at least once within 3 months after May 1, 2015. A lapse for these prevalence patients was any period 3 months or more between May 1, 2015, and April 30, 2016. To further understand lapse behavior for CCC patients, we also recorded whether the patient returned to the clinic after lapsing, but corresponding data for HIV clinic patients were unavailable.

### Data Analysis

Analysis of patients lapsing from the CCC and HIV clinics focused on clinic data collected between May 2015 and April 2016, yielding the following outcomes: the total number of patients in care; the number of new enrollees over 1 year; proportions of patients lapsed from care for 3 months from the CCC clinic and 2 months from the HIV clinic; the proportion of CCC patients that eventually returned to the CCC clinic.

Analysis of patients LTFU focused on the outcomes of community follow-up over a 1-year period, either from January through December 2016 or from November 2015 through October 2016. These outcomes included the proportion of patients located by the follow-up team and referred back to care; the proportion who refused to return back to care or who were unable to return; the proportion of confirmed deaths; the proportion of located patients erroneously designated as LTFU; the proportion of patients LTFU who reengaged in care; and the proportion of patients remaining in care 6 months after returning (for CCC patients, defined as 1 visit within 6 months after the initial return visit and 1 visit any time after 6 months).

Due to the different definitions of lapsed from care and LTFU and variable time intervals of data collection, the total number of patients for similar categories may differ between tables.

For statistical comparison of outcomes between feeder clinics and between new versus established patients, since *all* patients seen in the clinic during the designated time intervals were incorporated into the analysis and group selection was not biased in a systematic manner, we used a 2-sample proportion test (alpha=.05) and report *P* values of interest. However, since patient selection was not random, outcome differences must be interpreted carefully.

We also present approximate costs of the follow-up program over 1 year.

### Ethical Approval

The study was approved by Kisoro District Hospital and the institutional review board of the Albert Einstein College of Medicine.

## RESULTS

### Lapses From Care for CCC and HIV Patients

In 2015, the CCC had 5,046 patient visits. Of the total visits, 38% were for treatment of hypertension, 28% for diabetes, 9% for both hypertension and diabetes, 8% for congestive heart failure, 5% for asthma, 3% for epilepsy, and 8% for other conditions including renal, hepatic, and other chronic diseases.

Between May 2015 and April 2016, 223 patients enrolled as new CCC patients (incidence cohort). Of these, 95 (43%) lapsed from the clinic for 3 months within 1 year post-enrollment (Table 2). Of the 441 CCC patients active as of May 2015 (prevalence cohort), 252 (57%) lapsed for 3 months over the subsequent year. The difference between the incidence and prevalence cohort was significant at *P*<.001.

**TABLE 2.** Lapses From Care for Chronic Care Clinic and HIV Clinic Patients,^a^ Kisoro District Hospital, Uganda, May 2015–April 2016

	New Patients^b^	Existing Patients^c^
**CCC patients, N**	**223**	**441**
No. (%) of CCC patients who lapsed from care^d^	95 (43)	252 (57)
No. (%) of lapsed CCC patients who later returned	29 (31)	141 (56)
**HIV clinic patients, N**	**361**	**1321**
No. (%) of HIV patients who lapsed from care	216 (60)	401 (30)

Abbreviation: CCC, Chronic Care Clinic.

a Lapse from care defined as 3 or more months since the last appointment for CCC patients and 2 or more months for HIV clinic patients.

b New patients (inception cohort) are those who first enrolled in the clinic between May 2015 and April 2016.

c Existing patients (prevalence cohort) are those who made at least 3 clinic visits before May 2015 with at least 1 visit between January and April 2015, or, if they first enrolled in early 2015, returning at least once within 3 months after May 1, 2015.

d Median lapse=6 months; longest lapse=19 months.

Since its inception in 2005, a total of 3,921 patients attended the HIV clinic and 2,565 were prescribed monthly antiretroviral (ARV) medications. Of the incidence cohort of 361 patients newly enrolled between May 2015 and April 2016 and on ARVs, 216 (60%) lapsed for 2 months, interrupting therapy within their first year. Of the prevalence cohort of 1,321 patients active as of May 2015, 401 (30%) lapsed for 2 months within the year (Table 2). The difference between the incidence and prevalence cohort was significant at *P*<.001.

### Lost to Follow-Up for TB, Malnutrition, and Ward Discharge Patients

In 2016, 3,766 patients in total were admitted to KDH (including medical, surgical, pediatric, and maternity wards), 2,545 of whom were admitted to the medicine ward. Of those admitted to the medicine ward, 185 (7%) adults were diagnosed with TB (56% were documented by acid-fast bacilli testing and 44% were diagnosed clinically and treated empirically). In total, 79 (43%) missed a drug refill appointment by 2 or more weeks, thus interrupting therapy and triggering follow-up (Table 3).

**TABLE 3.** TB, Nutrition, and Ward Discharges LTFU, 2016^a^

	TB	Nutrition	Ward Discharges
Total number of new enrollees in 2016	185	245	448^b^
No. (%) of new enrollees LTFU	79 (43)	75 (31)	182 (41)

Abbreviations: LTFU, lost to follow-up; TB, tuberculosis.

a LTFU defined differently by hospital unit: TB=missed drug refill by 2 or more weeks; nutrition=missed 1 appointment; ward discharges=missed first CCC appointment by 1 month.

b 2,545 were admitted to the internal medicine ward in 2016 but only 448 were given follow-up appointments to the CCC upon discharge.

From 2008 to 2016, the nutrition clinic enrolled 3,067 severely malnourished patients. Of the 245 children enrolled in 2016, 75 (31%) missed at least 1 monthly appointment for food and monitoring.

In 2016, 2,545 patients were admitted to the internal medicine wards at KDH, with 448 given follow-up appointments to the CCC after discharge. Of these, 182 (41%) did not return within 1 month of their follow-up appointment.

### Follow-Up Outreach Outcomes

Over a 1-year period from November 2015 through October 2016, contact was attempted with 1,285 patients reported as LTFU. Table 4 details the outcomes of these attempts, per feeder. Of the total reported as LTFU, 816 (64%) were located in the community, whereas 469 (36%) could not be located. Of those located, 65% were referred to care (53% to a KDH-based clinic and 12% to another closer clinic), 19% had died, and 14% were not actually LTFU (listed erroneously). Only about 3% refused or were unable to return because they were imprisoned or bed-bound. Of those who could not be located, the follow-up team found that 36% had actually moved away from Kisoro.

Over a 1-year period in 2015–2016, 1,285 patients were reported as lost to follow-up, and the hospital's follow-up program located 64% of them.

**TABLE 4.** Follow-Up Outcomes Among Patients Lost to Follow-Up, by Hospital Unit, November 2015–October 2016 (N=1,285)

	CCC (n=310)	Ward Discharge (n=149)	HIV (n=691)	TB (n=73)	Nutrition (n=62)	Total (N= 1,285)
**Patients found, No. (%)**	**234 (75)**	**121(81)**	**360 (52)**	**54 (74)**	**47 (76)**	**816 (64)**
Recording error (*not* LTFU), No. (%)	39 (17)	11 (9)	57 (16)	4 (7)	4 (9)	115 (14)
Referred back to KDH clinic, No. (%)	142 (61)	81 (67)	138 (38)	36 (67)	32 (68)	429 (53)
Referred to another clinic, No. (%)	10 (4)	2 (1)	84 (23)	1 (2)	1 (2)	98 (12)
Refused to return, No. (%)	2 (1)	1 (1)	8 (2)	1 (2)	1 (2)	13 (2)
Unable to return (imprisoned, bed-bound), No. (%)	2 (1)	2 (1)	4 (1)	0 (0)	0 (0)	8 (1)
Confirmed dead	39 (17)	24 (20)	69 (19)	12 (22)	9 (19)	153 (19)
**Patients not found, No. (%)**	**76 (25)**	**28 (19)**	**331 (48)**	**19 (26)**	**15 (24)**	**469 (36)**
Not at home, No. (%)	9 (12)	3 (11)	9 (2)	0 (0)	1 (7)	22 (5)
Could not find home, No. (%)	32 (42)	15 (54)	214 (65)	10 (53)	8 (53)	279 (59)
Moved from Kisoro, No. (%)	35 (46)	10 (36)	108 (33)	9 (47)	6 (40)	168 (36)

Abbreviations: CCC, Chronic Care Clinic; KDH, Kisoro District Hospital; TB, tuberculosis.

Of note, the proportion of patients with HIV who were located (52%) was less than all other 4 feeder groups (range 74% to 81%, mean 77%; *P*<.001).

#### Reengagement in Care

Table 5 presents data on patient reengagement in care from the chronic disease feeders (lifelong therapy), comprising established CCC attendees, recent ward discharges with CCC appointments, and HIV clinic patients. As mentioned previously, active engagement in care at 6 months was defined as 1 visit within 6 months after the initial return visit and 1 visit any time after 6 months.

**TABLE 5.** Patient Reengagement Outcomes Among Patients With Chronic (Lifelong) Conditions Who Were Located and Referred Back to KDH, November 2015–October 2016 (N=361)

	CCC (n=142)	Ward Discharge (n=81)	HIV (n=138)
**Did not return to care, No. (%)**	**36 (25)**	**22 (27)**	**64 (46)**
**Returned to care, No. (%)**	**106 (75)**	**59 (73)**	**74 (54)**
6-month analysis not possible,^a^ No. (%)	18 (17)^b^	19 (32)^b^	11 (15)^b^
**Alive and eligible for 6-month follow-up, No. (%)**	**88 (83)**	**40 (68)**	**63 (85)**
Still in clinic at 6 months, No. (%)	62 (70)	21 (52)	43 (68)

Abbreviations: CCC, Chronic Care Condition; KDH, Kisoro District Hospital; LTFU, lost to follow-up.

a Analysis not possible because either the patient file was lost or the patient died before the 6-month mark, was discharged from the clinic, or was transferred to another clinic after returning.

b No. of patients who died before the 6-month analysis period: CCC (4), ward discharge (1), HIV (0), total (5).

Of the 459 CCC-related follow-up patients (CCC=310, ward discharges with CCC appointments=149), 223 (49%) were located and referred back to the CCC. Of those referred, 165 (74%) actually returned. This proportion was identical for both established CCC patients and ward discharges. However, the long-term result differed between these 2 CCC feeders. Of the 106 *established* CCC patients who returned, 88 (83%) were alive and in the district 6 months later and of these, 62 (70%) were still engaged with the CCC. A smaller proportion of ward discharge patients were alive or eligible for follow-up at 6 months (68%), and of these a smaller proportion again (52%) remained engaged with the CCC (*P*=.03 for proportion of eligible established CCC patients vs. ward discharges who remained engaged in care at 6 months).

Patients with HIV, who were harder to locate in the community, also returned to care less frequently than CCC-related patients after referral. Of the 138 patients with HIV who were located and referred back to the HIV clinic, only 74 (54%) returned (*P*<.001 when compared with CCC-related patients). However, if the patient returned once, the proportion who stayed in care for 6 months or more was similar regardless of whether the patient was a CCC-related patient (70%) or a patient with HIV (68%).

Table 6 presents data on patient reengagement in care from the TB and nutrition feeders (curative treatments). For the TB patients, 33 of the 36 (92%) patients located and referred to KDH returned after referral. For the malnourished patients, 23 of the 32 (72%) patients returned after referral.

**TABLE 6.** Patient Reengagement Outcomes Among Patients Receiving Curative Treatment Who Were Located and Referred Back to KDH, November 2015–October 2016 (N=68)

	TB (n=36)	Nutrition (n=32)
**Did not return to care, No. (%)**	3 (8)	9 (28)
**Returned to care, No. (%)**	33 (92)	23 (72)
Completed therapy, No. (%)	14 (42)	16 (70)
Still on therapy at time of analysis, No. (%)	3 (9)	3 (13)
Referred for treatment at a closer health center after returning, No. (%)	4 (12)	--
Refused treatment after returning, No. (%)	3 (9)	--
Died after returning, No. (%)	5 (15)	1 (4)
LTFU again, No. (%)	2 (6)	3 (13)
Charts lost and long-term outcome analysis not possible, No. (%)	2 (6)	0 (0)

Abbreviations: KDH, Kisoro District Hospital; LTFU, lost to follow-up; TB, tuberculosis.

Only 3 of the 36 TB patients located in the community and referred back to care failed to return. Of the 33 TB patients who initially returned, 2 charts were later lost, 3 patients refused further treatment, 10 became LTFU a second time before ultimately returning and reengaging in care after a second outreach, 2 were LTFU a third time and never completed treatment, and 5 died. However, 21 (64%) were alive and successfully reengaged in care: 14 completed treatment and 3 were still on treatment at KDH at the time of writing, and 4 had been transferred to closer health centers to complete therapy.

Similar to the TB patients, about half the malnourished children LTFU could be located in the community by the follow-up team and referred back to care. Of the 32 that were found and referred back to the nutrition clinic at KDH, 23 (72%) returned. Of the 23 who returned, 1 died, 3 were LTFU a third time, and 19 were successfully reengaged in the nutrition program. Of those reengaging in the nutrition program, 16 completed and 3 were completing treatment at the time of writing.

### Cost of the Program

In 2016, the total cost of the KDH follow-up program was approximately 23.8 million Ugandan shillings (US$6,600). Most of this cost—17.7 million Ugandan shillings (about US$4,900)—went either to salaries of full-time staff or program-related “top offs” of part-time staff primarily employed by KDH or the district. These costs do not include the services of U.S.-based consultant staff.

The performance-based point system increased staff income costs but more than tripled program productivity. With performance measured by points and facilitated by searching within geographical sets for a minimum of 8 patients, each staff could potentially earn 160% of his usual salary. In practice, the average outreach garnered 115% of the staff's per diem salary.

The next highest annual cost was for the motorcycles (including fuel, repair, and replacement but not amortized purchase cost of the motorcycles) used for transportation, averaging 4.9 million Ugandan shillings annually (US$1400), followed by miscellaneous costs (e.g., phone, Internet, office supplies) at $400.

## DISCUSSION

The myriad challenges of ensuring continuity of care in rural Africa involve patients, providers, and systems. Patient barriers include poverty, difficulties with understanding disease states and the importance of treatment and follow-up, and lack of access to health services. Provider barriers include inadequate training, inexperience, and turnover while systems-level barriers consist of understaffing, underfunding, drug stock-outs, donor mandates that may conflict with local hospital priorities, and lack of feasible strategies to support patients in continuous care. The Kisoro District Hospital implemented a follow-up program in an attempt to effectively and efficiently improve the continuity of care of a diverse range of patients.

**Figure:**
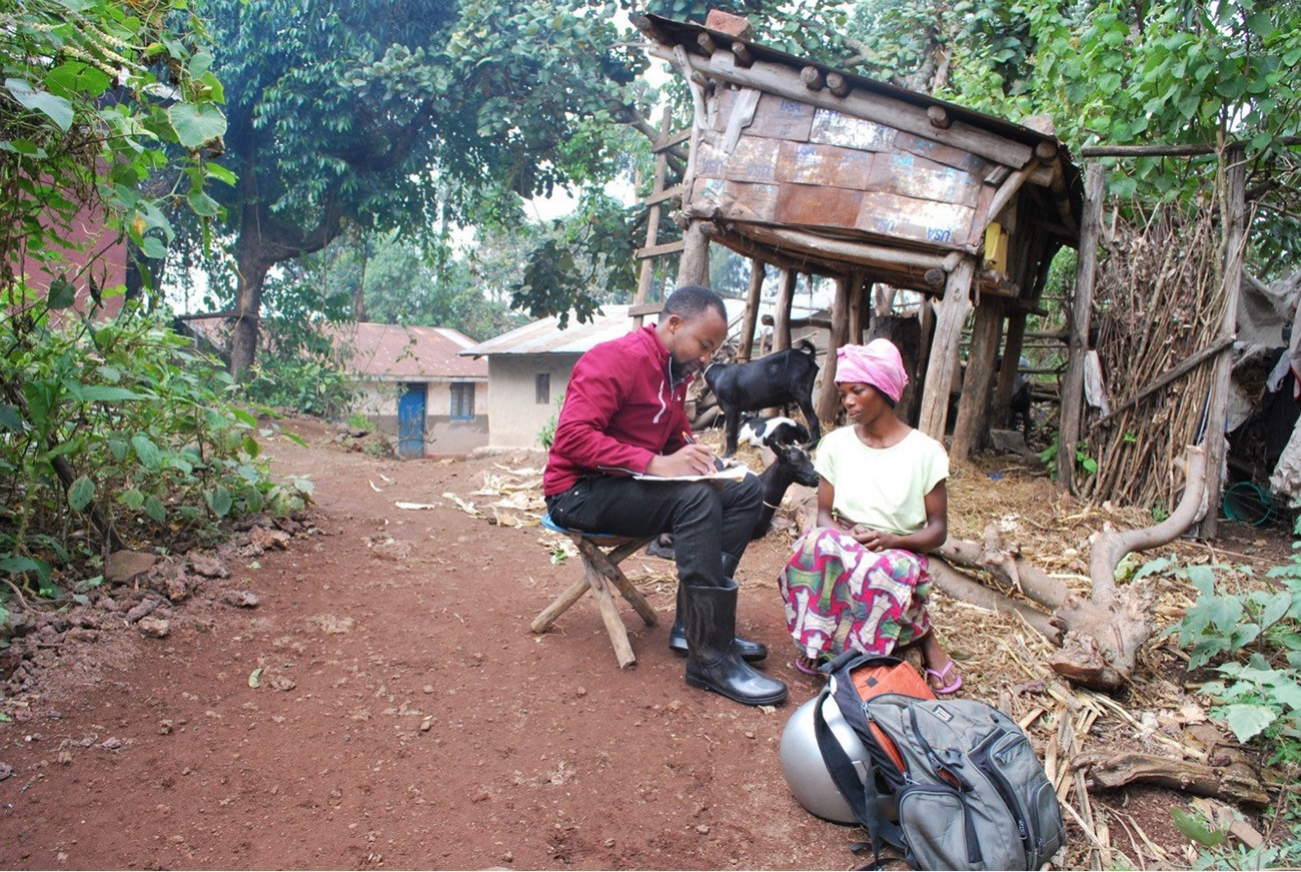
A field worker (author Gideon Muhoza) locates and meets with a patient who was lost to follow-up. © 2019 Julius Maniriho/Kisoro District Hospital

Almost all prior reports of follow-up activities have been “vertical” or disease-specific in nature, such as national TB or HIV programs, involving communicable diseases with significant public health impact.^3^^,^^13^^,^^18^^–^^22^ Employing telephone calls and home visits through outreach teams or community health workers, they showed moderate impact. For example, a 2013 systematic review of HIV clinics concluded that those that employed physical outreach had a lower LTFU rate (8%) than those using phone contact only (15%).^22^ There are few models employing follow-up approaches across multiple clinical domains. One example is South Africa's chronic disease management model, which integrates patients with NCDs, HIV, and TB in a common clinic and trained volunteers look for them if they lapse from care. Outcomes of the follow-up effort of this integrated program have yet to be published.^24^

In this article, we describe a “horizontal” strategy to maintain in care patients from various outpatient clinics of a remote, rural district hospital in Kisoro, Uganda. In a given geographical region, the number of patients LTFU from a full array of hospital-based clinical services will far outnumber patients from any one clinic, resulting in far greater yield of finding patients LTFU and potentially of cost-effectiveness of the program.

Interruption of therapy that is required over the long term or for life is a clinical challenge worldwide and is particularly evident in our rural African district. At KDH, across all clinics over a year, 30% to 60% of patients lapse or interrupt therapy for a clinically significant period, peaking at 60% for patients newly diagnosed with HIV. The magnitude of the issue is significant and similar for each disease or clinical source of patients–including TB (43% LTFU), malnutrition (31% LTFU), ward discharges (41% LTFU), NCD (43% and 57% of new and existing CCC patients, respectively, LTFU), and HIV (30% to 60% of existing and new HIV clinic patients, respectively, LTFU). Broad themes likely underpin the tendency of patients to drop out, such as poverty, distance, education, denial, unfriendly health systems, and “human nature”—themes that must be addressed systemically and socially.

At Kisoro District Hospital, across all clinics over 1 year, 30% to 60% of patients lapse or interrupt therapy for a clinically significant period.

The lapse from care data from the CCC documents the reality of patient adherence with monthly clinic appointments in rural Uganda, both for an “inception cohort” of new patients and a “prevalence cohort” of long-term patients. That about half of the long-term clinic patients with chronic disease lapse from the clinic for more than 3 months (median 5–6 months), with about half of these patients returning on their own, shows that long lapses from care are common and often temporary, at least where stock-outs are frequent and monthly visits are required to refill medications. To be cost-effective, follow-up programs should take this into account and establish appropriate lapse intervals and severity (or other clinically relevant) criteria before tracking patients.

Of interest, new enrollees lapse with frequencies quite different from long-term patients, and HIV and NCD (CCC) patients manifest opposite patterns. New enrollees with chronic diseases treated in the CCC are less likely than long-term patients to lapse for 3 months within the year (43% vs. 57%, respectively; *P*<.001), but if and when they do lapse, they are also less likely to return to clinic on their own (31% vs. 56%, respectively). The pattern for HIV patients was *opposite* that of CCC patients: 60% of new enrollees lapsed for 2 months versus 30% of long-term patients (*P*<.001).

We speculate that the differences in lapse rates between new and existing patients, and between feeder clinics, reflect diverse factors influencing patient behavior, such as diagnosis following clinical symptoms versus asymptomatic screening, social stigma, denial vs. acceptance, age, mobility, and sense of autonomy. For example, the above differences between the incidence and prevalence cohorts from the CCC may reflect a common experience of patients with chronic diseases: long-term patients lapse more frequently than new enrollees because they have seen that catastrophe is not immediate if they are non-adherent for a period, but they spontaneously return with greater frequency after a lapse because they generally believe in the merits of taking medication. Their long-term CCC enrollment selects for and reflects this response. The differences in follow-up behavior between new and long-term CCC patients highlight the risk inherent in drawing comparisons between different follow-up initiatives in different populations. At the systems level, distinct criteria and definitions of eligibility between feeder clinics and organizational shortcomings of hospital-based clinics are other potential explanations for observed differences.

Moving from hospital to community, the overall outcomes of the KDH follow-up program's find-and-engage strategy were relatively similar across “feeders” for patients with NCDs, TB, and malnutrition. Roughly 75% to 80% of patients LTFU could be located in the community. Of those located, about 20% had died and 65% were given a referral back to KDH. Of those referred, 70% to 75% actually returned (with the exception of TB patients, 92% of whom returned), and of those who returned, about two-thirds were still engaged in care 6 months later or completed therapy. These are gratifying results.

However, proportions are significantly different for patients with HIV. Patients with HIV proved harder to locate in the field, 52% HIV vs. about 77% other (*P*<.001), and when referred back to KDH were less likely to return, 54% HIV vs. 74% CCC and ward discharges (*P*<.001). The difficulty finding patients living with HIV in the community is undoubtedly multifactorial: such patients are young and “on the move,” often working outside Kisoro; are less likely to be known in the community than an elder with an NCD; may go by locally familiar nicknames unknown to the follow-up team; and, if from Rwanda or Congo, each 7 km from Kisoro, or if afraid of stigma, may even have registered with a false address. Once successfully contacted, their lower likelihood of returning to the clinic could well involve denial of their HIV diagnosis, especially if the diagnosis was recent and health temporarily restored by treatment. Stigma/denial as an explanation for those who did not return is consistent with the observation that those who did return were just as likely to stay in care (68%) as those from other clinics.

Of note, patients undergoing treatment for TB could be located as frequently in the community as those with NCDs (about 75%) but once found and referred, TB patients were much more likely to return for medication (92% TB vs. 74% CCC and ward discharges; *P*=.03). This is not surprising given the policy mandate to treat TB, backed by the threat of forced confinement if necessary, and the very short lapse (2 weeks) triggering an active search. On the other hand, that 43% of patients with TB became LTFU (by our stricter definition) and 26% of these could not be located or had moved from the district highlights the importance of sound systems of interdistrict communication and tracing patients until treatment completion. The observation that of the 33 patients with TB who returned initially, 10 were LTFU a second time before reengaging in care after a second outreach highlights the importance (and expense) of maintaining adherence with TB treatment. Although the numbers are small, the “granular” TB data from our follow-up program call into question the accuracy of national reports from low-income countries of TB treatment success of approximately 80% of higher. (In 2017, WHO reported Uganda's treatment success rate to be 77%.^25^)

The percentage (19%) of patients confirmed dead by the follow-up team and the recording errors that identified active patients as LTFU (14%) total 33% and add considerably to the cost of follow-up efforts without improving health. The high mortality of rural African patients implies that deaths be accurately tallied when assessing long-term adherence with care. Although there is epidemiologic value in documenting mortality, the errors in accurately linking charts with patients focuses attention on the basic infrastructure required before longitudinal care can become maximally cost-effective in low-income countries.

Only 1% of patients LTFU and contacted refused to return, but 25% of CCC and 45% of HIV patients seen in the community and referred to KDH never returned. This begs the question of whether the patients appreciated being contacted by hospital personnel and whether the program's approach of presumptive consent on the part of patients to be contacted was in fact the most appropriate approach. Preliminary data from surveys of both ward and clinic patients reveal that about 95% of surveyed patients are comfortable with and welcome the follow-up initiative. In the future, we will be soliciting informed consent from patients enrolled in our feeder clinics ahead of time to allow future community-based follow-up in case of lapses in care or loss to follow-up.

What will this follow-up model look like in the future? Although KDH will not use electronic medical records anytime soon, in 2019 we anticipate identifying eligible LTFU patients via an electronic appointment registry for all CCC, ward discharge, HIV, and women's clinic patients, and thereafter, TB patients. We are preparing systems that will identify patients automatically incorporating disease severity (and thus follow-up priority), phone numbers when available, and the village/“set” of the patient's home. Important additional features such as applying patient identifiers to help trace HIV and TB patients when they transfer sites will have to await government initiatives in these arenas for consistency and cohesion.

Kisoro District Hospital is currently undergoing preparations to identify eligible patients lost to follow-up through an electronic system.

Steps are also being taken in the CCC to improve service and thereby limit lapses from care, including streamlining the appointment system; providing drug refills more readily for suitable patients; and, as more families gain phone access, implementing a call service to save patients the time and expense of making appointments in person.

Although a formal cost-effectiveness analysis was not performed, the tallied costs of the program for 1 year was approximately US$6,600, amounting to US$5 per patient designated as LTFU and US$40 per patient found, reengaged, and completed or continued on therapy. With more efficient and accurate electronic identification of patients eligible for follow-up, these per person costs could decrease substantially. It is likely that for communicable and/or treatable diseases, including TB, HIV, and malnutrition, these costs, though considerable in the context of the miniscule health budgets of many African countries, are worth it to contain disease spread and improve workforce productivity. (The Ugandan per capita overall health expenditure annually was about US$40 to $50 between 2013 and 2016, with the government supporting less than 20% and out-of-pocket expenses totaling about 40%.^26^) For NCDs, the picture is not as clear, and a long-term lens that focuses on the financial implications for both the individual and caregiving families of the prevention of complications like stroke, heart disease, and renal failure, would have to be adopted.

The cost of the hospital's follow-up program amounts to US$5/patient designated as lost to follow-up and $40/patient found and reengaged.

### Limitations

Several limitations must be acknowledged. First, the article is a retrospective description of an ongoing program, started more than 6 years ago, whose objectives were not research, but service. Second, the definitions of LTFU vary between clinics, and despite maximizing clinical relevance and feasibility for the clinic for the most part they do not conform to similar definitions in the literature. Likewise, the data were recorded by myriad providers of care and were input by clinic staff with less consistency than in a prospective study. This last issue also led to the use of slightly different annual time frames to describe different data sets, skirting months with lost data or unrepresentative personnel changes.

Even if the approach described herein is adopted, results may vary in other settings. Attitudes and practices related to chronic diseases are influenced by education level and local myths and beliefs. They vary between countries, regions within a country, and cultures. Adherence with appointments and therefore frequency of LTFU are affected by medication stock-outs, appointment frequency, distance from and access to the clinic, provider skill and familiarity, clinic function, and options for care elsewhere. Seemingly small details can affect follow up, e.g., the CCC enrolled patients only after they returned to the clinic at least once, thereby selecting for a more adherent patient population rather than an “inception cohort” of “all-comers.” In addition, many of the CCC's providers have been Western volunteers.

## CONCLUSION

Despite the local realities of care, the “horizontal,” hospital-wide follow-up program approach of following up with patients from diverse hospital clinics and wards is novel, feasible under circumstances such as those found at Kisoro District Hospital, and maximally efficient in rural settings. The program has been operational for more than 6 years and is well integrated into the function of the hospital. Its organization contrasts with follow-up programs that are disease-specific or “vertical,” with each clinical service following only patients with one defined (and separately funded) disease or health issue.

Four key features of an effective multi-service follow-up program in this setting include:

- Application of clinically relevant criteria for triggering follow-up of LTFU patients, devised in partnership with feeder clinics
- Employing a distinct and dedicated team of follow-up staff who is familiar with the communities, has experience with inquiring about patient whereabouts while maintaining confidentiality, and is committed to meeting regularly with clinic personnel
- Organization of villages according to “geographical sets” served by common roads, with outreach triggered by a minimum number of patients to locate per set from multiple feeders
- Stipends for staff based on productivity

The outcomes of the KDH follow-up program have been quite positive, although for reasons discussed, reengaging patients with HIV who were LTFU has proven most challenging. In general, of patients without HIV infection, about 75% to 80% LTFU could be located in the community, 70% to 75% of those referred back to KDH actually returned, and of those who returned about two-thirds were either still engaged in care 6 months later or completed therapy.

## Notes

**Peer Reviewed**

**Funding:** This work was supported by the Global Health Center of the Albert Einstein College of Medicine.
